# Constitutive activated STAT3 is an essential regulator and therapeutic target in esophageal squamous cell carcinoma

**DOI:** 10.18632/oncotarget.20838

**Published:** 2017-09-12

**Authors:** Fang Tian, Xiawen Yang, Ying Liu, Xiao Yuan, Tianli Fan, Fanmiao Zhang, Jimin Zhao, Jing Lu, Yanan Jiang, Ziming Dong, Yili Yang

**Affiliations:** ^1^ Department of Pathophysiology, School of Basic Medical Sciences, Zhengzhou University, Zhengzhou, Henan, P. R. China; ^2^ Henan Provincial Cooperative Innovation Center for Cancer Chemoprevention, Zhengzhou, Henan, P. R. China; ^3^ Suzhou Institute of Systems Medicine, Center for Systems Medicine, Chinese Academy of Medical Sciences, Suzhou, Jiangsu, P. R. China; ^4^ Division of Molecular Signaling, Department of Advanced Biomedical Research, University of Yamanashi, Yamanashi, Japan

**Keywords:** esophageal squamous cell carcinoma, STAT3, apoptosis, patient-derived xenograft, targeting therapy

## Abstract

Esophageal carcinoma is among the most common cancers worldwide and a leading cause of cancer death [1]. Large numbers of studies indicated that chronic inflammation is closely associated with its development [21, 25]. Furthermore, the JAK/STAT pathway, which plays a critical role in inflammation and immunity, has been implied in a number of malignancies [11]. It has been shown that targeting the pathway affected the growth, apoptosis, and metastasis of cultured esophageal squamous cell carcinoma cells [26]. We found in the present study that STAT3 is constitutively activated in a subgroup of esophageal squamous cell carcinoma cell lines and primary tumors. Altered expressions of STAT3 target genes were found in these tumors by using RNAseq and qPCR analysis. Cytokines that activate STAT3 affected the expression of STAT3 target genes and promoted the growth of the ESCC cells, which could be blocked by STAT3 inhibitor and specific siRNA. Inhibition of STAT3 also suppressed the growth and colony formation, and induced apoptosis in the esophageal squamous cell carcinoma cells containing constitutively activated STAT3. Furthermore, the STAT3 inhibitor effectively blocked the growth of patient-derived tumor xenografts that harbored phosphorylated STAT3, but acted less effective on the xenografts derived from primary tumors that contained low levels of activated STAT3. These results indicated that activated STAT3 plays a critical role in the survival and growth of a subgroup of esophageal squamous cell carcinoma, and may serve as a target for precision therapeutic intervention.

## INTRODUCTION

Esophageal cancer is one of the most common malignancies that causes the death of more than 400,000 people annually worldwide [1]. The majority of esophageal cancers are squamous cell carcinoma (ESCC), up to 90% in some investigations. Large number of studies indicated that the development of ESCC is closely associated with genetic susceptibility, environmental factors, and chronic inflammatory [2]. However, despite significant progress in finding genomic, molecular, and metabolic changes in esophageal cancers [26], the 5-year survival rates of patients with ESCC remain very low are (less than 30%) [3].

The JAK-STAT pathway mediates the signaling of many growth factors and cytokines, which uses the STAT family transcription factors (STAT1, 2, and 3) to regulate the expression of a diverse set of target genes [4], and affects profoundly cell growth, differentiation, and apoptosis [5]. It also plays a critical role in inflammation, innate and adaptive immunity, and involved in the development of human disorders, including autoimmune diseases and cancers [6]. Noteworthily, STAT3 has been found closely associated with various human cancers [7, 8, 9, 10]. Aberrant activated STAT3 contributes to cell transformation, angiogenesis, metastasis, immune evasion, and epithelial-mesenchymal transition [27, 28]. The increased activation of STAT3 also promotes invasion of ESCC cells. Thus, STAT3 has been an actively sought-after target for anti-cancer drug discovery, and a number of specific inhibitors have been developed for further clinical studies [11].

In this study, we found that a subgroup of primary ESCCs and ESCC cell lines expressed high level constitutively activated STAT3 and exhibited STAT3-regulated gene expression profiles. While STAT3-activating cytokines promoted the growth of ESCC cells, inhibition of STAT3 suppressed their growth and colony formation. Furthermore, ESCC patients-derived xenografts containing high levels of pSTAT3 were sensitive to STAT3 inhibitors, whereas these expressed low levels of pSTAT3 were relatively resistant. These data indicate that constitutively activated STAT3 plays a critical role in ESCC and targeting STAT3 is a potential effective therapeutic strategy for the cancer.

## RESULTS

### Activation of STAT3 in esophageal squamous cell cancers

**Figure 1:**
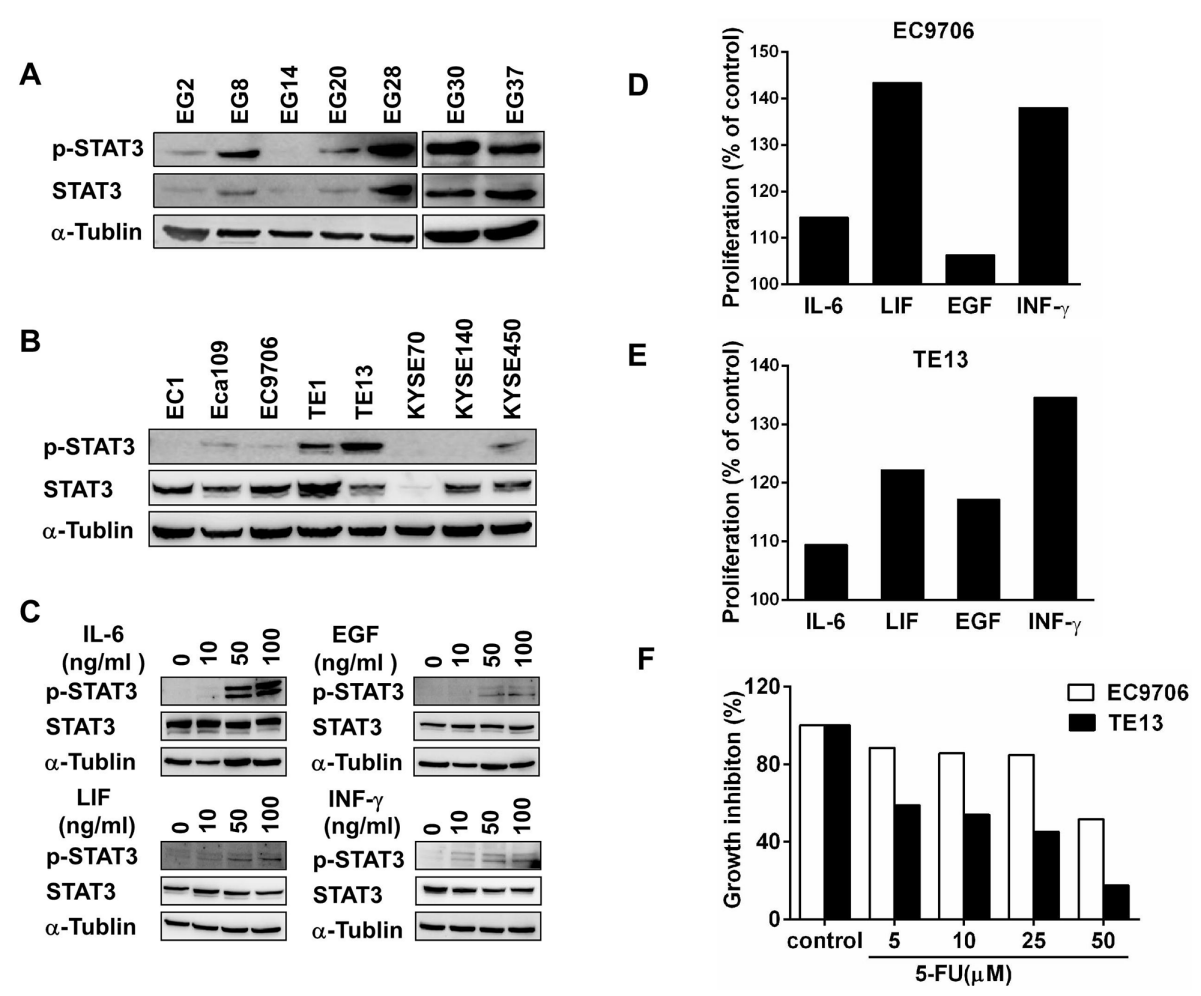
STAT3 is activated in a subgroup of esophageal squamous cell cancers **(A)** Expression of STAT3 and pSTAT3 in primary ESCC samples from 8 patients were examined by immunoblotting. **(B)** Expression of STAT3 and pSTAT3 in 8 different lines of ESCC cells were examined by immunoblotting. **(C)** EC9706 cells were treated with indicated doses of cytokines and growth factors for 30 minutes. They were harvest to assess the expression of STAT3 and p-STAT3. **(D)** and **(E)** The proliferation of EC9706 and TE13 were examined by using CCK-8 after treated with 10 ng/ml of IL-6, LIF, EGF, or INF-γ for 48h. Cells cultured with medium alone were regarded as 100%. **(F)** After treated with indicated doses of 5-FU, the growth of EC9706 and TE13 cells were examined by using CCK-8.

The close association between inflammation and esophageal cancers prompted us to examine the expression of STAT3 and its activation in primary esophageal squamous cell carcinomas. Among the 7 tumors samples from patients with ESCC, 6 of them clearly expressed STAT3 and p-STAT3 (Tyr-705) when examined by immunoblotting (Figure 1A). Although the level of STAT3 and pSTAT3 varied significantly, specimens EG8, EG28, EG30, and EG37 constantly exhibited high level of pSTAT3, indicating that STAT3 was constitutively activated in these tumors. The clinicopathological features of these patients were summarized in Supplementary Table 1.

To further explore the role of activated STAT3 in esophageal cancers, 8 different ESCC cell lines were examined by immunoblotting using the anti-STAT3 and anti-phosphorylated STAT3 (Tyr-705) antibodies. While most of the cells expressed STAT3, TE1 and TE13 contained high level of phosphorylated STAT3, and Eca109, EC9706, and KYSE450 had relative low but detectable levels of pSTAT3 (Figure 1B). We then asked whether the JAK-STAT3 pathway could be activated by cytokines and EGF in these cells. In EC9706 cells, IL-6, EGF, LIF and INF-γall activated STAT3 in a dose-dependent manner, although the level of total STAT3 was not increased during the 30-minutes treatment (Figure 1C). These cytokines and EGF also affected the growth of EC9706 and TE13 cells significantly (Figure 1D & 1E). Thus, STAT3 is constitutively activated in certain ESCC cells. The STAT3 pathway in ESCC cells can be activated by growth factor and inflammatory cytokines, affecting their growth and drug sensitivity.

### Stattic inhibited STAT3 activation in ESCC cells

**Figure 2:**
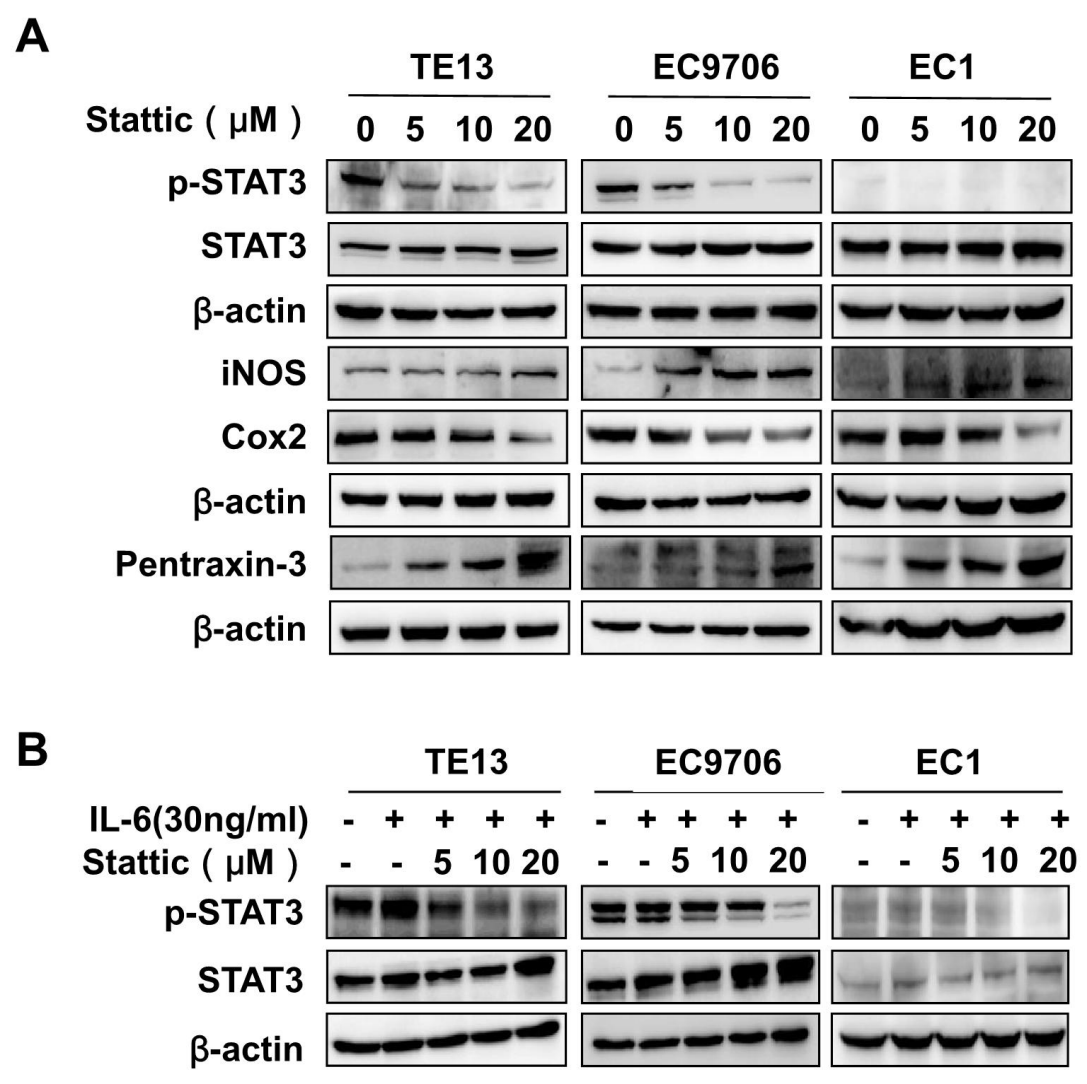
Stattic inhibited STAT3 activation in ESCC cells **(A)** TE13, EC9706 and EC1 cells were treated with indicated concentrations of Stattic. The expression of STAT3, p-STAT3, iNOS, Cox2, and Pentraxin-3 were examined by immunoblotting with specific antibodies. The level of β-actin was used as a control. **(B)** EC9706, TE13 and EC1 cells were exposed to 30 ng/ml of IL-6 in the presence of indicated concentrations of Stattic. The levels of STAT3 and pSTAT3 were examined by immunoblotting.

It has been shown that small molecular STAT3 inhibitor Stattic is cell permeable and can block the phosphorylation of tyrosine in the SH2 domain of STAT3 and inhibit the binding of dimerized STAT3 to specific DNA response element [12]. We therefore examined its effects on TE13, EC9706, and EC1 cells, which express high, medium, and undetectable levels of phosphorylated STAT3 respectively (Figure 1B). As shown in Figure 2A, Stattic decreased the levels of p-STAT3, but not that of total STAT3, in EC9706 and TE13 cells dose-dependently. The expression of inflammation and immunity-related Cox2, iNOS, and pentraxin-3 were then assessed to gauge the effects of Stattic. While the level of Cox2, upregulated by activated STAT3 in the cells [15], is decreased in TE13 and EC9706 upon Stattic treatment, the level of iNOS and pentraxin-3, whose expression were negatively regulated by p-STAT3 [16, 17], were increased in Stattic-treated cells. However, the expression of these genes did not change significantly in EC1 cells, which did not contain notable pSTAT3 under these conditions. Stattic also effectively blocked IL-6-induced STAT3 activation in TE13 and EC9706 cells (Figure 2B).

### Constitutively activated STAT3 is required for esophageal squamous cancer cells growth and survival

**Figure 3:**
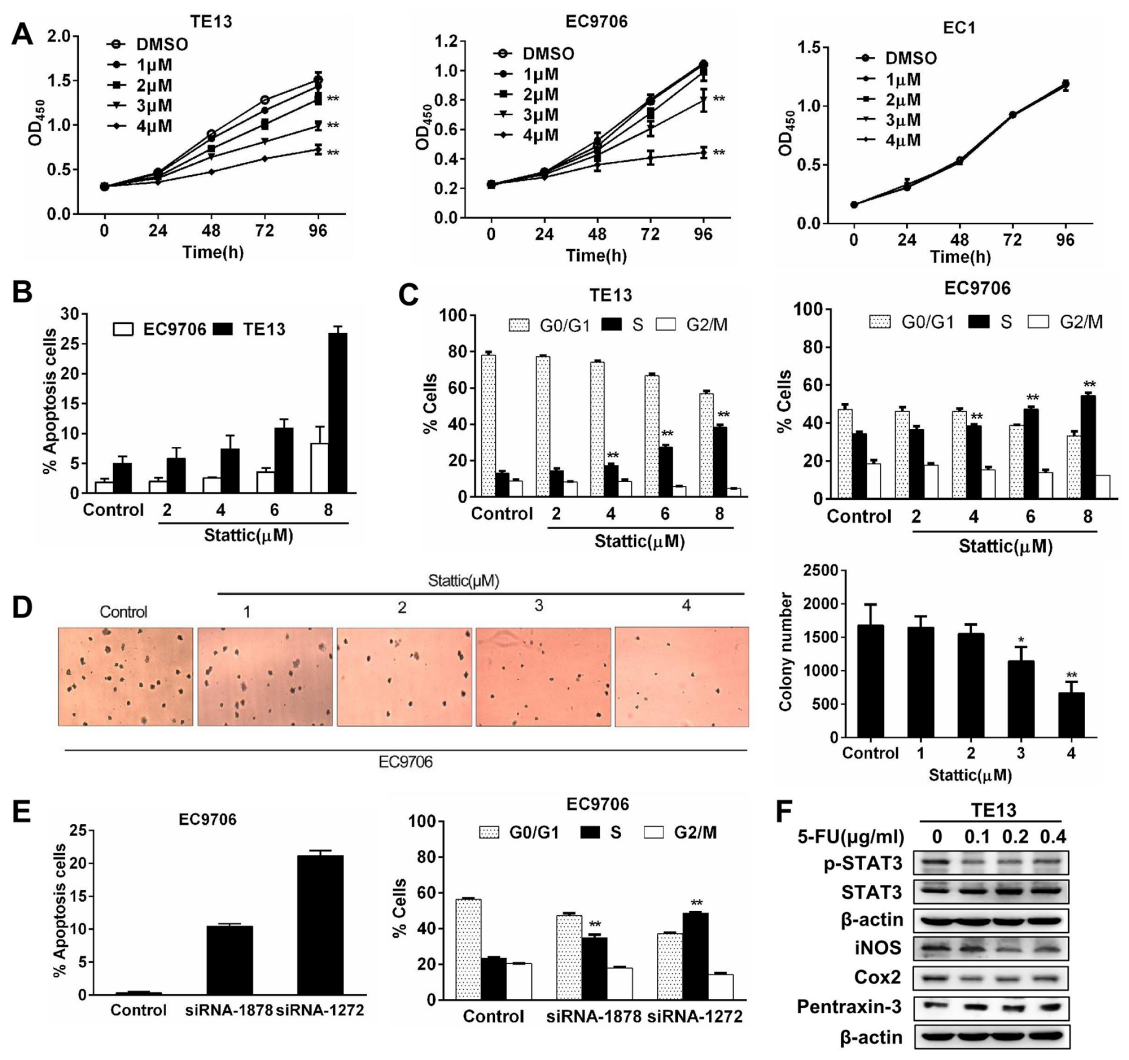
Constitutively activated STAT3 is required for the growth and survival of esophageal squamous cancer cells **(A)** The growth of EC9706, TE13, and EC1 cells in the presence of Stattic. At 72h and 96h, 1 - 4 μM of Stattic significantly inhibited the growth of TE13 and EC9706 cells, but not that of EC1 (**p<0.01). **(B)** Stattic dose-dependently induced apoptosis in EC9706 and TE13 cells. Data were expressed as means±SD from three independent experiments. **(C)** Stattic treatment led to S phase blockage in EC9706 and TE13. Data were means of three independent experiments. **(D)** Stattic dose-dependently reduced colony formation from EC9706 cells. Colony formation assay using soft agar was performed. Colonies were counted under a microscope and the data were expressed as means±SD from three different experiments (*p<0.05, **p<0.01). **(E)** Two STAT3-trageting siRNAs induced apoptosis and cell cycle blockage in EC9706 cells. **(F)** Treatment of TE3 cells with 5-FU led to decreased pSTAT3 and altered expression of iNOS, Cox2, and pentraxin-3.

To understand the role of activated STAT3 in ESCC cells, the growth of EC9706, TE13 and EC1 cells were assessed in the presence of Stattic. Low doses of Stattic (1-4μM) induced a dose-dependent slow down of growth in EC9706 and TE13 cells, but not that of EC1 cells (Figure 3A). When higher doses of Stattic were used, they induced significant growth inhibition in EC9706 and TE13 cells. We examined apoptosis in Stattic-treated cells by using annexin V staining. While 6 and 8 μM of Stattic increased apoptosis moderately in EC9706 cells, they induced significant more apoptosis in TE13 cells, which contained higher level of pSTAT3 (Figure 3B). Cell cycle analysis revealed that Stattic treatment led to a decrease of cells in G1 phase and a S phase blockage in the cells (Figure 3C). In soft agar assay of EC9706 cells, 3 and 4 μM of Stattic decreased the numbers of colony formation by 22 and 55% respectively (Figure 3D). To further determine the specificity of the effects of Stattic treatment, we transfected EC9706 cells with two siRNAs targeting STAT3 (siRNA-1878 and siRNA-1272). As shown in Figure 3E, they also caused S phase cell cycle block and apoptosis in the cells. Consistent with these findings, 5-FU, which kill ESCC cells effectively, reduced the level of pSTAT3, but not that of total STAT3 in TE13 cells. Reduction of pSTAT3 was also accompanied by the alteration of STAT3-regulated iNOS, Cox2, and pentraxin (Figure 3F). Taken together, these results indicated that activated STAT3 is required for these ESCC cells to growth and survive, and for their tumorigenic action.

### Blocking STAT3 signaling suppresses tumor growth in PDX model

**Figure 4:**
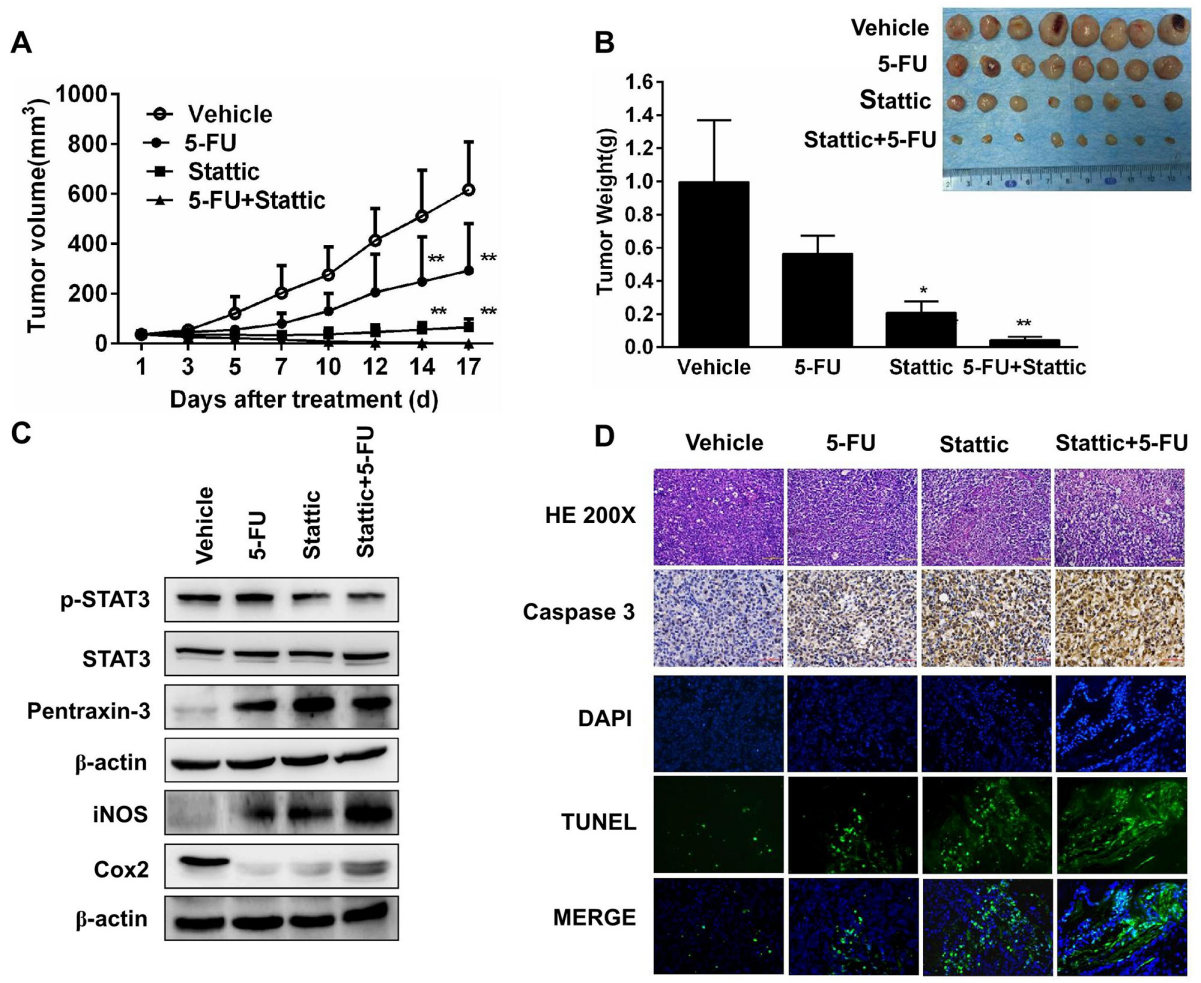
Xenografs from primary tumor EG30, which contained high level of pSTAT3, was sensitive to STAT3 inhibition **(A)** 8 Mice bearing PDX were treated with vehicle, 5-FU, Stattic, or 5-FU+Stattic. While 5-FU alone inhibited tumor growth moderately, Stattic alone significantly inhibited the xenografts growth (**P<0.01 compared with vehicle group). **(B)** Photograph of tumors from the 4 groups at the end of the experiments. Weight of the xenografts were measured (*p<0.05, **p<0.01 compared with vehicle group). **(C)** Immunoblotting of STAT3, pSTAT3, pentraxin-3, iNOS, and Cox2 in the xenografts. Stattic markedly decreased pSTAT3 level and altered the levels of pentraxin-3, iNOS, and Cox2. **(D)** Histological and immunohistochemical analysis of the xenografts. Compared with control, xenografts from Stattic and Stattic plus 5-FU-treated mice contained higher levels of activated caspase-3 and TUNEL^+^ cells.

To further examine whether activation of STAT3 is essential for ESCC cells to grow and form tumor, we established patients-derived xenografts in SCID mice from primary tumors EG30 and EG37, which contained high level pSTAT3, and EG2 and EG14 that expressed low level of phosphorylated STAT3. Mice with the xenografts were treated with the vehicle, 5-FU, Stattic, or Stattic plus 5-FU. As shown in Figure 4A, Stattic alone significantly inhibited the growth of xenografts derived from EG30. After these mice were sacrificed by the end of the 2^nd^ week, the volume of the tumors from mice received Stattic were less than one fourth of that from mice given the vehicle (Figure 4B). 5-FU also had notable effect in inhibiting the growth of the xenografts, although it appeared significantly less effective compared with Stattic under these experimental conditions. Noteworthily, Stattic and 5-FU together had strong synergistic anti-tumor action, leading to significant greater tumor growth inhibition. We also found that animals received vehicle developed anorexia and weight loss in the second week, whereas Stattic and 5-FU treated mice did not have any notable symptoms.

We furthermore analyzed tumors from mice treated with these agents. Phosphorylated STAT3 in tumors from mice administrated with Stattic and Stattic plus 5-FU was significantly reduced, and the levels of pSTAT3-regulated proteins, Cox2, iNOS, and Pentraxin-3 were all markedly altered accordingly (Figure 4C). HE and anti-caspase 3 staining indicated that there were less cell proliferation and significantly increased apoptosis in tumors from mice received Stattic alone or together with 5-FU. The increased apoptosis in Stattic-treated tumors was further revealed by the TUNEL assay (Figure 4D).

**Figure 5:**
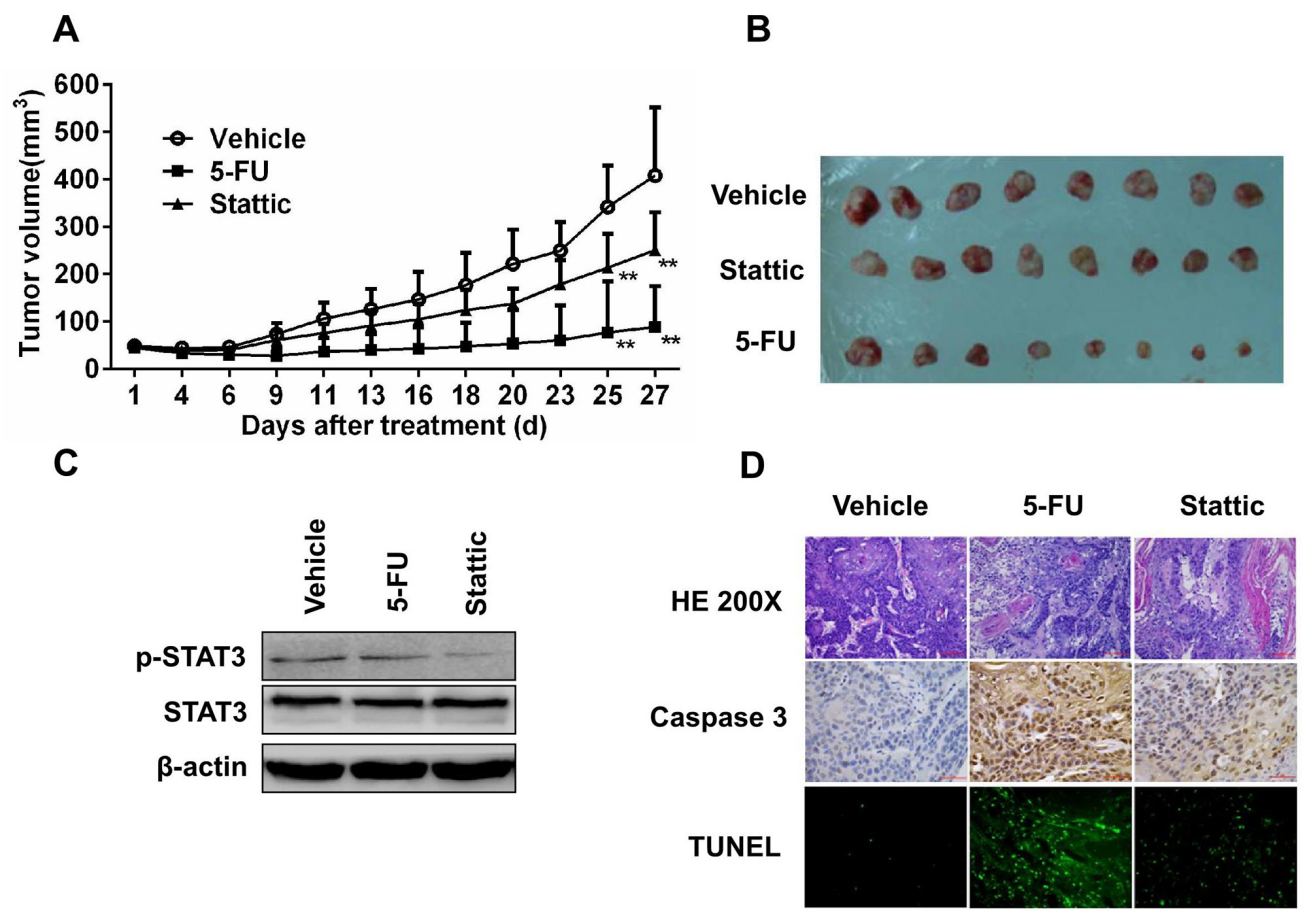
Xenografts from primary tumor EG2, which contained low level of pSTAT3, was relative insensitive to STAT3 inhibition **(A)** 8 Mice bearing the PDX were treated with vehicle, 5-FU, or Stattic. While 5-FU alone inhibited tumor growth significantly, Stattic alone only decreased the xenografts growth slightly (**P<0.01 compared with vehicle group). **(B)** Photograph of tumors from the 3 groups at the end of the experiments. **(C)** Immunoblotting of STAT3 and pSTAT3 in the xenografts. **(D)** Histological and immunohistochemical analysis of the xenografts. Compared with control, xenografts from 5-FU-treated mice contained higher levels of activated caspase-3 and TUNEL+ cells, whereas were insignificant in these from Stattic-treated mice.

Similar results were found when PDX generated from primary tumor EG37 were exposed to various therapeutics *in vivo* and analyzed *in vitro* (Supplementary Figure 1). Interestingly, while PDX from primary tumors EG30 and EG37 were more sensitive to Stattic than 5-FU, xenografts derived from primary tumors EG2 and EG14 were significantly more sensitive to 5-FU, and were only moderately susceptible to the inhibitory action of Stattic (Figure 5A & 5B and Supplementary Figure 2A & 2B). In these tumor tissues, pSTAT3 level was low and only slightly reduced by Stattic (Figure 5C and Supplementary Figure 2C). Immunohistochemical analysis showed that 5-FU induced a significant increase of activated caspase-3 and TUNEL-positive cells, whereas the increases induced by Stattic were more moderate (Figure 5D and Supplementary Figure 2D). Taken together, these data demonstrated that activated STAT3 is required for the growth and survival of the tumors that contained high level of the protein, and targeting pSTAT3 could be an effective strategy for the treatment of these tumors.

### Differential expressed STAT3 target genes in pSTAT3-high xenograft

**Figure 6:**
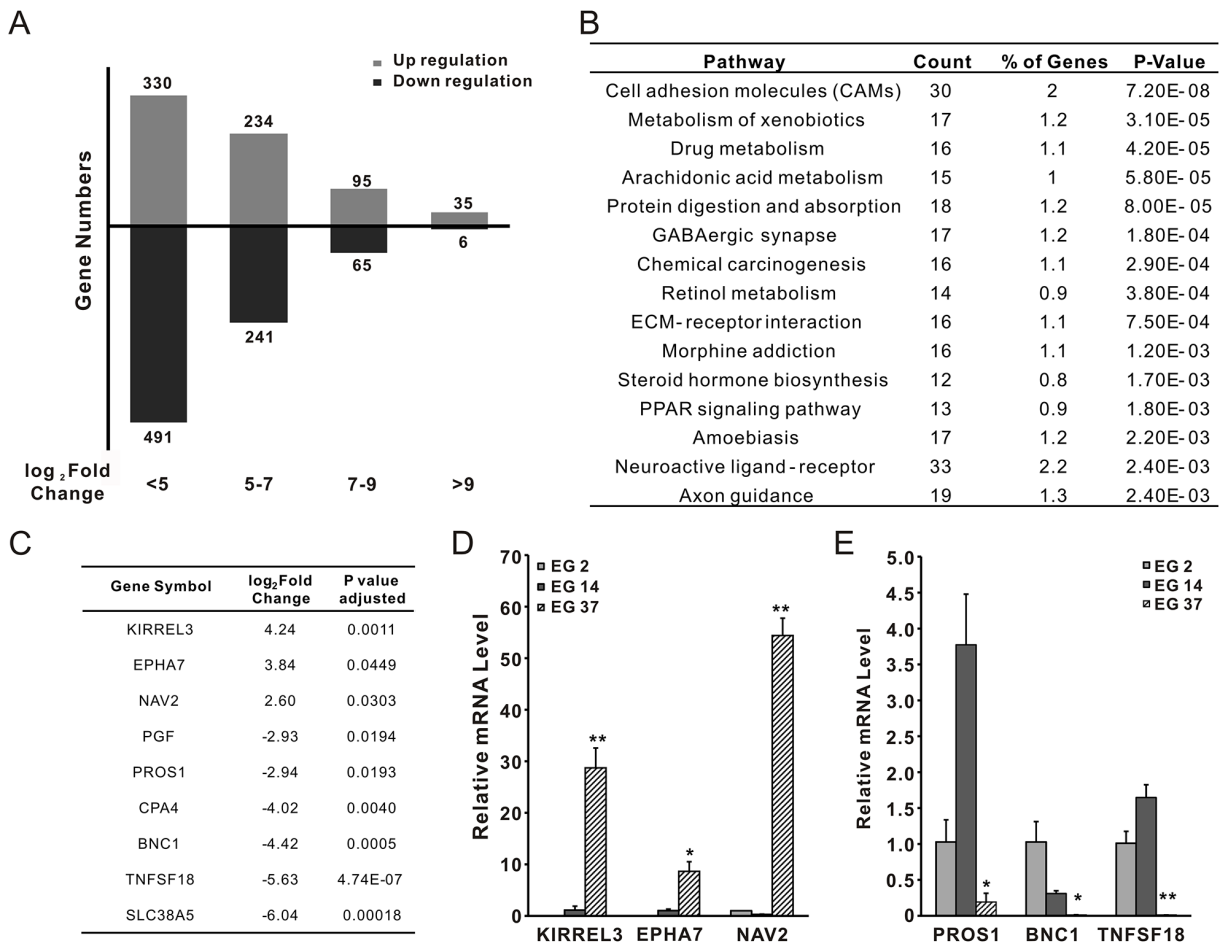
RNAseq analysis of xenografts from primary tumors EG2, EG14, and EG37 **(A)** Differential expressed genes between pSTAT3-high xenografts from EG37 and pSTAT3-low xenografts from EG2 and EG14. **(B)** Based on the KEGG database, the altered genes was in a number of cellular pathways, most notably the cell adhesion molecules. **(C)** 9 of the altered genes contained putative STAT3 binding site in their promote regions. **(D)** and **(E)** qPCR confirmed lower expression of BNC1, TNFSF18, and PROS1, and higher expression of NAV2, KIRRET3, EPHA7 in xenograft from EG37, when compared with that of xenografts from EG2 and EG14.

To further understand the role of activated STAT3 in ESCC, RNAseq was successfully performed with xenografts derived from EG2, EG14, and EG37. Compared with the gene expression profiles of EG2 and EG14, we found that the expression of 1497 genes was altered more than 5 folds, among which, 803 were upregulated, and 694 were downregulated (Figure 6A). Noteworthily, kinases PI3 (>9 folds) and RIPK3 (>7 folds) were among the most significantly elevated genes, suggesting that they might associate with the increased STAT3 phosphorylation in EG37 tumors. According to KEGG database, the altered genes were significantly involved in a number of pathways, in particularly the cell adhesion molecules (Figure 6B). We also found that 9 of the altered genes contained putative STAT3 binding site (http://software.broadinstitute.org/gsea/msigdb/cards/V$STAT3_02) in their promote regions (Figure 6C). To further confirm the results, qPCR was used to assess the expression of the genes NAV2, KIRRET3, EPHA7, BNC1, TNFSF18, and PROS1 in the xenografts. Compared with that of EG2 and EG14, the expression of NAV2, KIRRET3, and EPHA7 was significantly elevated in xenografts of EG37 (Figure 6D), whereas that of BNC1, TNFSF18, and PROS1 was significantly decreased (Figure 6E). Therefore, pSTAT3 in the xenografts is likely functioning through regulating the expression of multiple target genes.

## DISCUSSION

Gastrointestinal malignancies are often associated with chronic inflammation. It has been well documented that inflammatory bowel diseases predispose to colorectal cancer, Barrett’s esophagus is a precursor of esophageal adenocarcinoma, *Helicobacter pylori*induces gastric cancer [18]. There are also evidences relating chronic inflammation with esophageal squamous cell carcinoma [19]. However, the molecular mechanisms link inflammation and cancers are still not well understood in most cases. It has been found that NFκB activation, through both classic and alternative pathways, plays a critical role in inflammation-associated gastrointestinal carcinogenesis [20]. It was also reported that chronic inflammation in ESCC is associated with the oxidative DNA damage and DNA double-strand breaks (DSBs) [21]. The level of oxidative DNA damage is positively correlated with the degree of chronic inflammation, which also has a positive correlation with esophageal precursor lesions, suggesting that inflammation may promote the development of ESCC through inducing genomic instability. COX-2 expression in ESCC was also found correlated with the degree of dysplasia, poor prognosis and chemotherapy resistance [22]. COX-2 inhibition led to decreased cell proliferation, PGE2 production and overall tumor progression *in vitro* and *in vivo*. Since STAT3 is a positive regulator of Cox2, it is interesting to further explore whether it mediates the role of STAT3 in ESSC. It should be noted that there are close connections between the STAT3 and NFκB pathways. One of the connection is bridged by IL-6, whose expression is upregulated by NFκB, and in turn activates STAT3 [23]. STAT3 and NFκB also share a number of downstream targets. Given the critical role of NFκB in cell growth and survival, it is conceivable that, in addition to cyclin D1 and c-Myc, NFκB may mediate the effects of STAT3 on esophageal cancers.

The implications of STAT3 in various cancers and in cancer initiation, development, metastasis, and drug resistance make it a desirable target for therapeutic target. A variety of STAT3 inhibitors, including peptide mimetics, oligonucleotides, and small molecules, have been developed [11, 24]. While peptide mimetics and oligonucleotides can be directed toward all 3 domains of STAT3, most of the small molecules act on the SH2 domain. Similar to what we found in the present study, it has also been shown that STAT3 inhibition in mice and human trials induced only moderate side effects in various cells and tissues, indicating that STAT3 is likely a suitable target for cancer therapy. However, no STAT3 inhibitors have reached the clinic, despite some promising preclinical studies and multiple clinical trials. Efforts are being made to increase the efficacy and specificity of small molecules STAT3 inhibitors that act on protein-protein interaction. It also raised the questions that whether STAT3 activation is indispensable for cancer cells to survive. However, in light of the results presented, it is conceivable that preclinical or clinical studies based on pSTAT3 and STAT3-regulated genes may facilitate the finding of effective drugs and strategies targeting the STAT3 pathway.

## MATERIALS AND METHODS

### Reagents

STAT3 inhibitor Stattic [12] was purchased from Sigma (St. Louis, MO). 5-FU was purchased from Xudong Haipu (Shanghai). Mouse monoclonal antibody against STAT3 (sc-9139); rabbit monoclonal antibody against Cox2 (sc-12282); rabbit polyclonal antibodies against Phospho-STAT3 (Tyr705) (sc-9131) and iNOS (sc-2977) were purchased from Cell Signaling Biotechnology (Beverly, MA). Rabbit monoclonal antibody against Pentraxin-3 was purchased from Abcam (Cambridge, MA). Mouse monoclonal antibodies against α-Tublin (sc-8035) and β-Actin (sc-47778) were purchased from Santa Cruz Biotechnology (Santa Cruz,CA). Annexin V-FITC Kit was purchased from Beyotime Biotechnology (Shanghai). The STAT3-targeting siRNAs, siRNA-STAT3-1272 (sc-82859), siRNA-STAT3-1878 (sc-82860), and scrambled control (sc-1511170) were purchased from GenePharma (Shanghai).

### Cell lines and cell culture

ESCC cell lines, EC1, Eca109, EC9706, KYSE 70 and KYSE 140 were cultured in RPMI 1640 supplemented with 10% fetal bovine serum (FBS) (Hyclone, UT), 100U/ml penicillin, and 100μg/ml streptomycin. TE1, TE13, KYSE 450 were cultured in DMEM supplemented with 10% FBS, 100U/ml penicillin, and 100μg/ml streptomycin. All of these cells were maintained at 37°C with 5% CO_2_.

### Colony formation assay

The colony formation assay was performed using soft agar as described {Puck, T. T., Marcus, P. I., Cieciura, S. J. J Exp Med, 1956)}. EC9706 cells (2.4×10^4^) mixed with top agar in Basal Medium Eagle supplemented with 10% FBS were seeded in a 6-well culture plate in the presence of indicated concentrations of Stattic. After 14 days, the colonies were counted under a microscope and pictures were token.

### Cell proliferation assay

Cells (7×10^3^/well) were in 96-well plates overnight, and then exposed to Stattic (0, 1, 2, 3, 4μM). After culturing for 0, 24, 48, 72 and 96 hours, 10μl of CCK8 reagent (Keygen, Nanjing) was added to the each well. Following incubation at 37°C for 1.5h, OD_450_ was measured as indication of cell proliferation.

### siRNA transfection

For immunoblotting analysis, 3×10^5^ EC9706 cells were seeded in a 6cm tissue culture dish and incubated overnight. They were transfected with 126 pmol of indicated siRNA for 24h. After changed to the complete medium, the cells were collected 12, 24 and 48 hours later respectively. For apoptosis and cell cycle assays, EC9706 cells (1.3×10^5^) were seeded into a 96-well plate and incubated overnight. After transfected with the medium containing siRNA (126 pmol), cells were cultured for 24h and collected for apoptosis and cell cycle analysis.

### Immunoblotting

Cell lysate was prepared by using modified-RIPA buffer (Tris 50mM, NP-40 1%, NaDOC 0.25%, NaCl 150mM, EDTA 1mM, SDS 0.1%, NaF 5mM, NaVO3 0.4 mM, and protease inhibitors). Tissue proteins were extracted with the tissue protein extraction reagent (Beyotime, Shanghai) supplemented with protease inhibitors cocktail. Protein was quantitated by using the BCA protein concentration kit (Beyotime). Samples were separated by using 10% SDS-PAGE. After transferred to a PVDF membrane, immunoblotting was carried out using various specific antibodies and visualized with enhanced chemiluminescence and the Image Reader LAS-4000 (Fuji, Japan).

### Apoptosis and cell cycle analysis

Apoptosis was detected using the Annexin V staining (Beyotime). Cell cycle was analyzed using PI staining and the In Cell Analyzer 2000 (GE health system, WI).

### ESCC tissues

Human esophageal squamous tumor specimens were collected from patients enrolled into the First Affiliates Hospital of Zhengzhou University and underwent tumorectomy after received informed consent and approved by the Ethics Committee of First Affiliates Hospital of Zhengzhou University. All specimens were examined and confirmed by pathologists. None of these patients had received preoperative chemotherapy or radiation therapy. Due to the priority was assigned to the pathological evaluation, only a limited amount of tissues were available from each tumors for investigation. The patients-derived xenografts were established as described [13], and the 3^rd^ passages were allowed to grow to ∼ 200 mm^3^ and used to carry out experiments described in the present studies.

### Xenograft tumor models

The animal studies were approved by the Ethics Committee of Basic Medical College of Zhengzhou University. SCID mice were purchased from the Beijing Weitonglihua (Beijing, China). The 4- to 6-week-old male mice were maintained in laminar flow cabinets under specific pathogen free conditions. The patients-derived xenografts were generated as described [13]. The 3^rd^ passages of PDX of EG2, EG8, EG30, and EG37 were used. The tumors were cut into small pieces (∼3 mm in diameter), implanted subcutaneously into a numbers of mice. They were then randomly divided into different treatment groups. EG2:(a) control vehicle (80% DMSO + 20% PBS); (b) 10mg/kg of Stattic; (c) 10mg/kg of 5-FU. EG14: (a) control vehicle (80% DMSO + 20% PBS); (b) 10mg/kg of 5-FU; (c) 10 mg/kg of Stattic; EG30 and EG37: (a) control vehicle (80% DMSO + 20% PBS); (b) 10mg/kg of 5-FU; (c) 10mg/kg of Stattic; (d)10mg/kg of Stattic with 10mg/kg of 5-FU. The compounds were administered via intraperitoneal injection three times a week. The tumors were measured in two dimensions with calipers ∼ every 3 days, and the volume was calculated as length × width^2^× 1/2.

### Immunohistochemical analysis

Immunohistochemical staining was carried out with sections of formalin-fixedparaffin-embedded tumor specimens. Antigen was retrievedin sodium citrate (0.01 mol/l, pH6.0). After incubated sequentially with anti-caspase 3 (1:50) and HRP-labeled secondary antibodies, the sections were stained with DAB and counter-stained with Hematoxylinand Eosin.

### TUNEL assay

DNA fragmentation in cells undergoing apoptosis was examined by the TUNEL assay. Briefly, tissue sections were deparaffinized in xylene, treated with a graded series of alcohol and distilled water, and washed thoroughly with PBS. After incubated with proteinase K (20 μg/ml in PBS) for 20 min at RT, terminal deoxynucleotidyl transferased UTP nick end-labeling (TUNEL) was carried out using the in situ cell death detection kit (KeyGen, Nanjing) according to the manufacturer’s instruction. TUNEL-positive cells from 5 independent fields were counted under microscope.

### RNAseq and data analysis

Total RNA was extracted from xenografts derived from EG2, EG14, EG30, and EG37 by using RNeasy Mini Kit (Qiagen) according to the manufacturer’s instruction, and was quantitated by using the Qubit 2.0 fluorometer. The quality of the RNA preparations was assessed using the Agilent Technology 2100 Bioanalyzer. Ribosomal RNA was removed by the Ribo-Zero rRNA removal kit (Epicenter, Madison, WI). The RNA libraries were constructed using a TruSeq Stranded RNA sample preparation kit (RS-122-2001, Illumina) according to the manufacturer’s protocol. After assessed with the Qubit and Bioanalyzer, their molar concentrations were validated by qPCR. Sequencing was performed on the Illumina HiSeq platform using PE150 chemistry by the sequencing core facility of Suzhou Institute of Systems Medicine. The average reads for the samples was 22G.

Differentially expressed transcripts were identified using the linear model implemented in R package ballgown [14]. Transcript FPKM was used as a measurement of transcript expression, and the fold change was calculated by comparing the mean of samples from EG2 and EG14 cells against the samples from EG37 xenograft. Genes expressed differentially between the groups were selected into the list based on P value < 0.05 (calculated by Ballgown’s F-test) and fold-change > 5.

### Statistical analysis

Statistical analysis of the results was performed by one-way analysis of variance (ANOVA), or Student *t* test using SPSS version 17.0 (SPSS, Chicago, IL). Results are expressed as means ± standard deviation, P<0.05 was considered as a significant.

## SUPPLEMENTARY MATERIALS FIGURES AND TABLE


