# Supplementary material for: Constitutive activated STAT3 is an essential regulator and therapeutic target in esophageal squamous cell carcinoma
Source: Oncotarget. 2017 Sep 12;8(51):88719–29. doi: 10.18632/oncotarget.20838 (PMC5687640; doi:10.18632/oncotarget.20838)
Supplement: Supplementary file 1 [file oncotarget-08-88719-s001.pdf]

Constitutive activated STAT3 is an essential regulator and therapeutic target in esophageal squamous cell carcinoma

SUPPLEMENTARY MATERIALS

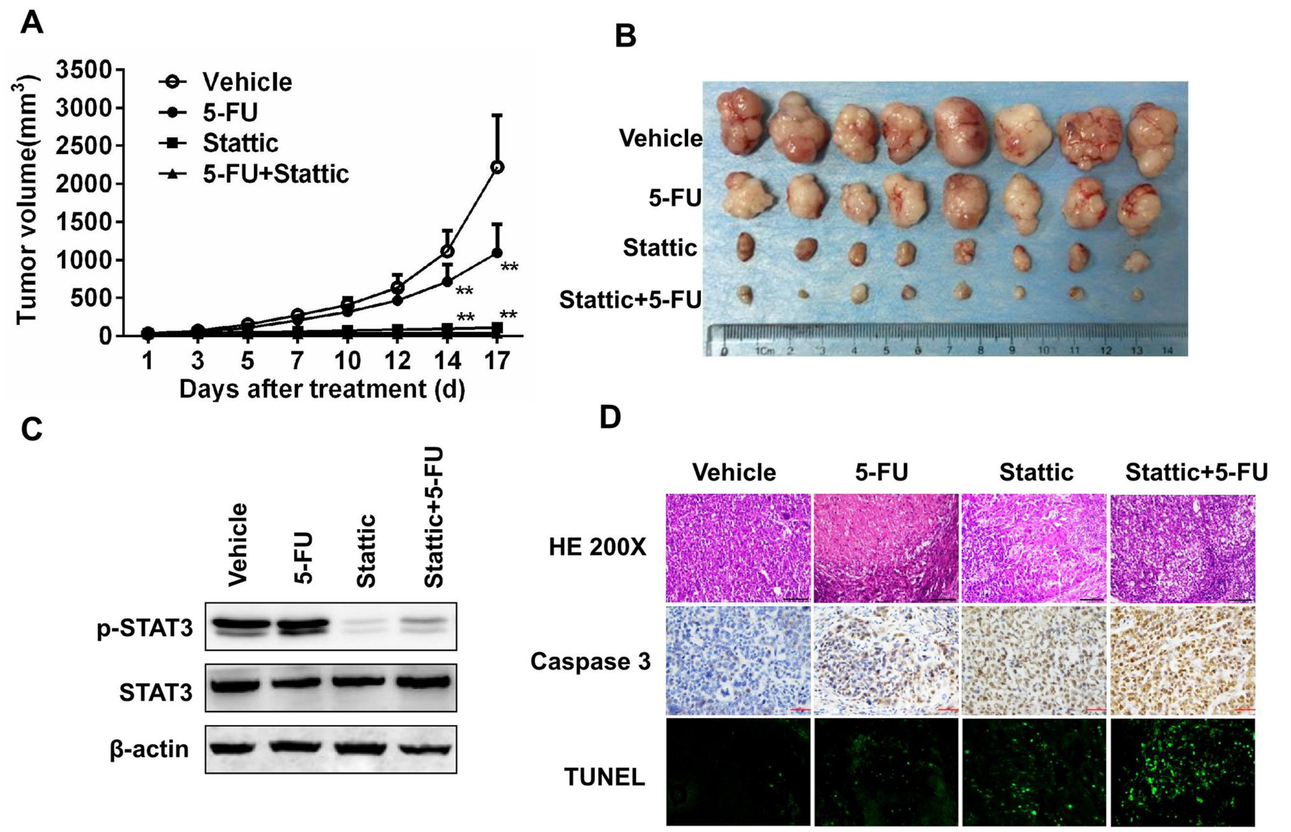

Supplementary Figure 1: Xenografs from primary tumor EG37, which contained high level of pSTAT3, was sensitive to STAT3 inhibition. **(A)** 8 Mice bearing PDX were treated with vehicle, 5-FU, Stattic, or 5FU+Stattic. While 5-FU alone inhibited tumor growth moderately, Stattic alone significantly inhibited the xenografts growth (**P<0.01 compared with vehicle group). **(B)** Photograph of tumors from the 4 groups at the end of the experiments. **(C)** Immunoblotting of STAT3 and pSTAT3, in the xenografts. Stattic markedly decreased pSTAT3 level. **(D)** Histological and immunohistochemical analysis of the xenografts. Compared with control, xenografts from Stattic and Stattic plus 5-FU-treated mice contained higher levels of activated caspase-3 and TUNEL$^+$ cells.

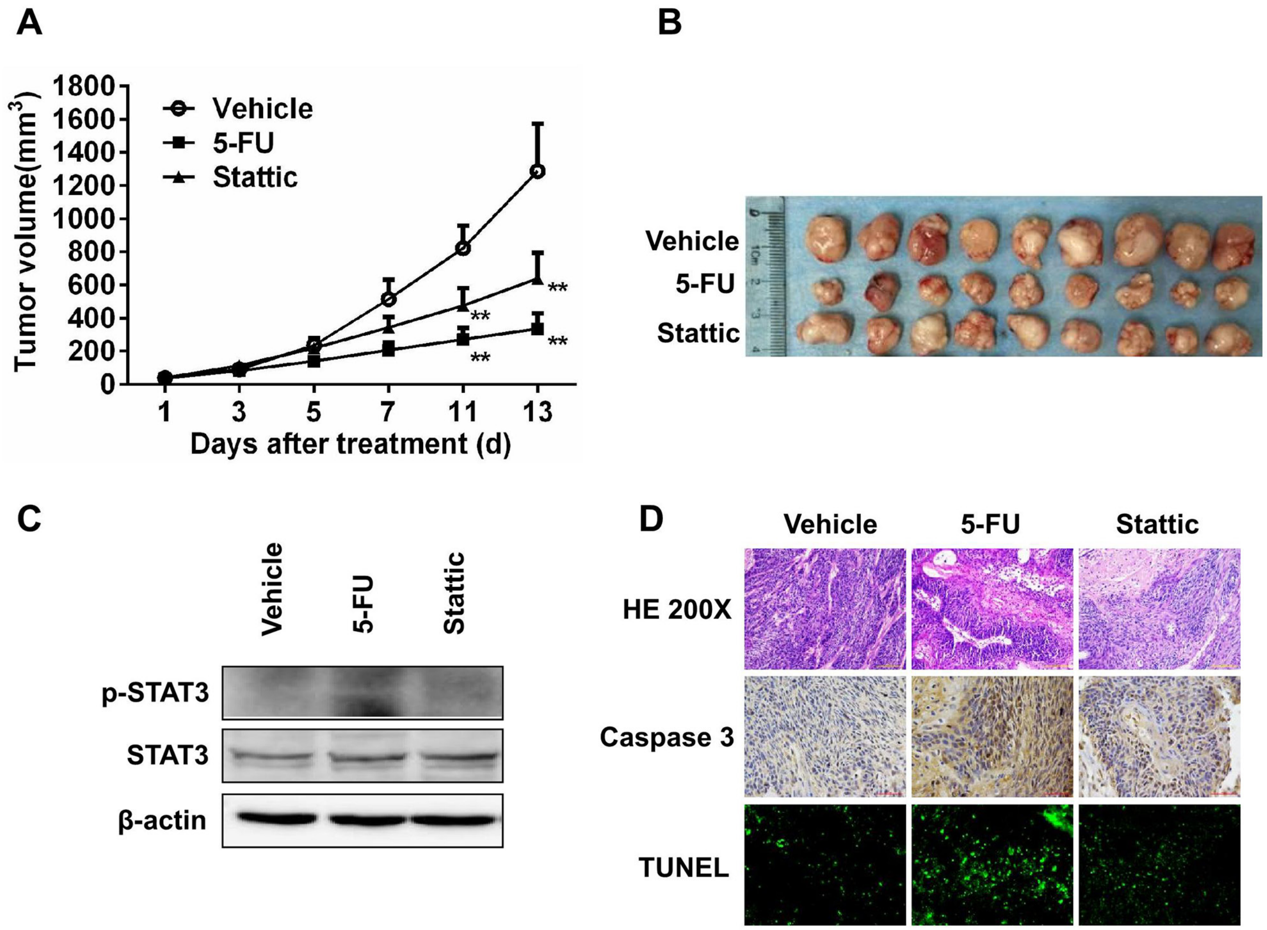

Supplementary Figure 2: Xenografts from primary tumor EG14, which contained low level of pSTAT3, was relative insensitive to STAT3 inhibition. (A) 8 Mice bearing the PDX were treated with vehicle, 5-FU, or Stattic. While 5-FU alone inhibited tumor growth significantly, Stattic alone only decreased the xenografts growth slightly (**P<0.01 compared with vehicle group). **(B)** Photograph of tumors from the 3 groups at the end of the experiments. **(C)** Immunoblotting of STAT3 and pSTAT3 in the xenografts. **(D)** Histological and immunohistochemical analysis of the xenografts. Compared with control, xenografts from 5-FU-treated mice contained higher levels of activated caspase-3 and TUNEL+ cells, whereas were insignificant in these from Stattic-treated mice.

Supplementary Table 1 : The clinicopathological patients with esophageal cancers.

Number	Gender	Age	Tumor	Type	TNM staging	Tumor differentiation
EG2	Male	64	Esophagus	ESCC	T2N0M0	II
EG8	Male	63	Esophagus	ESCC	T2N0M0	II
EG14	Male	59	Esophagus	ESCC	T2N0M0	II
EG20	Female	46	Esophagus	ESCC	T2N0M0	II
EG28	Male	60	Esophagus	ESCC	T2N1M0	II
EG30	Male	73	Esophagus	ESCC	T3N0M0	II
EG37	Male	69	Esophagus	ESCC	T3N0M0	II

ESCC: esophageal squamous cell carcinoma.
T: tumor; N: lymph nodes; M: metastasis.
